# Working Memory Updating Training Improves Mathematics Performance in Middle School Students With Learning Difficulties

**DOI:** 10.3389/fnhum.2018.00154

**Published:** 2018-04-24

**Authors:** Hongxia Zhang, Lei Chang, Xiaoying Chen, Liang Ma, Renlai Zhou

**Affiliations:** ^1^Faculty of Psychology, Beijing Normal University, Beijing, China; ^2^Department of Psychology, University of Macau, Macau, China; ^3^Department of Psychology, Nanjing University, Nanjing, China

**Keywords:** learning difficulties, working memory, updating training, N160, P300

## Abstract

Working memory (WM) deficit is considered the key cause of learning difficulties (LDs). Studies have shown that WM is plastic and thus can be improved through training. This positive effect is transferable to fluid intelligence and academic performance. This study investigated whether WM updating ability and academic performance in children with LDs could be improved through WM updating training and explored the effects of this training on the children’s brain activity. We used a running memory task lasting approximately 40 min per day for 28 days to train a group of 23 children with LDs (TLDs group). We also selected two control groups of 22 children with LDs (CLDs group) and 20 children without LDs (normal control [NC] group). The behavioral results of a pretest indicated that WM updating ability and academic performance in the TLDs and CLDs groups were significantly lower than those in the NC group before training. Compared with the CLDs group, the TLDs group exhibited significant performance improvement in a 2-back WM task, as well as in mathematical ability. Event-related potentials (ERPs) results suggested that the amplitudes of N160 (representative of visual recognition) and P300 (representative of updating processing, which is a valid index for updating WM) in the TLDs and CLDs groups were markedly lower than those in the NC group before training. In the TLDs group, these two components increased considerably after training, approaching levels similar to those in the NC group. The results of this study suggest that WM updating training can improve WM updating ability in children with LDs and the training effect can transfer to mathematical performance in such children. Furthermore, the participants’ brain activity levels can exhibit positive changes. This article provides experimental evidence that WM updating training could mitigate the symptoms of LDs to a certain degree.

## Introduction

Learning difficulties (LDs) refer to deficiencies in an individual’s capacity to acquire the skills required for learning reading, writing and arithmetic to a level comparable to that of other people of similar age, education and intelligence. Although LDs are not directly caused by motivational, emotional, or attentional problems, such problems are typically considered comorbidities (Hammill, 1990). The three most common types of LDs are difficulties in reading (Gathercole et al., 2006), mathematics (Geary et al., 2007), and a combination of the two (Swanson and Beebe-Frankenberger, 2004). The prevalence of LDs is substantial among school children, affecting 10%–15% (Hendriksen et al., 2007), thereby imposing considerable economic and mental burdens on society and families. Researchers urgently need to determine the exact pathogenesis of LDs to enable educators to explore intervention methods.

Although numerous researchers in various fields have devoted themselves to exploring the pathogenesis of LDs, it remains unclear. In recent years, working memory (WM) deficit has been regarded as a critical contributing factor of LDs. Although different types of LDs in children are related to different cognitive defects (Peng and Fuchs, 2014), LDs generally involve WM deficits. Numerous studies have indicated that WM capability is the most frequently impaired function in children with LDs (Gathercole et al., 2006; Maehler and Schuchardt, 2009; Pimperton and Nation, 2010; De Weerdt et al., 2013; Peng and Fuchs, 2014). WM refers to the ability to temporarily maintain and manipulate information while performing cognitive tasks (Baddeley, 1992). Compared with short-term memory, WM plays a more influential role in children’s academic performance because many academic tasks involve multiple steps with intermediate solutions that children need to remember as they proceed (McKenzie et al., 2003; Cain et al., 2004). According to Baddeley’s Multicomponent Model that WM consists of three components: the central executive, the phonological loop and the visuospatial sketchpad (Baddeley, 1992, 2003). The central executive, which is the core of WM, is responsible for coordinating the slave systems (phonological loop and visuospatial sketchpad), focusing and switching attention, and retrieving representations from long-term memory, as well as for updating, inhibiting and shifting. The results of several studies have indicated that WM impairment in individuals with LDs is caused by impairment of the central executive, particularly that of the updating aspect of executive functions (Carretti et al., 2005; Alloway et al., 2006; Wang et al., 2011; Zhao et al., 2011, 2013). Functional magnetic resonance imaging (fMRI) studies based on neuroscience research have suggested that the connectivity of the frontoparietal network, which is responsible for storing and processing information, is weaker in people with LDs than in those without them (Rotzer et al., 2009; Koyama et al., 2013). The event-related potential (ERP) technique features high resolution in a time course and can distinguish between changes in different stages of WM processing based on temporal activation patterns. ERP studies have indicated that the amplitudes of the P300 component which is representative of updating processing is considerably attenuated in people with LDs compared with those without them (Dainer et al., 1981; Taylor and Keenan, 1990).

Previous studies have suggested that WM capability is plastic, and thus can be improved through WM updating training, particularly in individuals with WM deficiencies (Klingberg et al., 2002, 2005; Jaeggi et al., 2008; Zhao et al., 2011; Rutledge et al., 2012; Sprenger et al., 2013; Chen et al., 2017). WM updating training generally involves employing computerized adaptive technology in a step-by-step manner to enable subjects to practice various WM tasks such as span and updating tasks. This technology can overcome the difficulty of automatically adjusting training tasks to suit a trainee’s performance, and thus has the potential to improve his or her WM capability to the greatest possible extent (Shipstead et al., 2010; Zhao and Zhou, 2010; Melby-Lervåg and Hulme, 2013).

Cognitive neuroscience studies regarding the effects of WM updating training have indicated that a trainee’s activity levels in various brain regions can be changed through the training process; for example, the amplitudes of the N160 and P300 components in adult trainees can increase substantially and the P200 amplitudes can decrease substantially through training (Hempel et al., 2004; Dahlin et al., 2008; Zhao et al., 2013). The N160 component can be interpreted as relevant in terms of perceptual speed and visual attention allocation (McEvoy et al., 2001; Zhao et al., 2013). The P300 component is an effective index for the updating function (Donchin and Coles, 1988; Gevins and Smith, 2000). The P200 component evoked by the frontal area reflects the inhibition of irrelevant information and the ability to attend to the target stimulus (Friedman et al., 1993; McEvoy et al., 2001; Zhao et al., 2013). However, for certain populations such as people with attention deficit hyperactivity disorder (ADHD), alcohol spectrum disorders, or stroke, or elderly adults whose cognitive functions have deteriorated, no concrete evidence suggests that WM updating training changed brain activity (Klingberg et al., 2002; Westerberg and Klingberg, 2007; Loomes et al., 2008).The results of previous studies have indicated not only that WM updating training can improve the WM capability of trainees but also that the effects of training can be transferred to other cognitive functions associated with WM such as fluid intelligence (Jaeggi et al., 2008), attention, reading ability (Chein and Morrison, 2010; Loosli et al., 2011), and mathematical ability (Holmes et al., 2009). Few studies have investigated WM training in relation to LDs, and those that have focused on cognitive behavioral changes after training. Although the results of these studies were not all the same, they all showed that the WM training could mitigate the symptoms of LDs to a certain degree (Zhong, 2011; Gray et al., 2012; Gropper et al., 2014; Chen et al., 2017). Furthermore, studies exploring neural mechanisms and changes in brain function are scarce.

The current study adopted three versions of adaptive running memory tasks as WM updating training tasks to investigate their effects on relieving the symptoms of LDs, which vary in terms of behavior and related brain activity, as indicated by their respective ERPs. The adaptive running memory task is widely used as an index of WM capability on updating. In this task, a series of unknown items of a certain length are presented to participants, then they are required to recall in order within a certain length of time. This task better represents the ability to monitor input information and to replace old information that is irrelevant to the ongoing task with new information that is relevant to the ongoing task (Morris and Jones, 1990). Based on the literature on LDs, WM, and the benefits of WM training, we predicted that WM updating training would not only increase WM capability in people with LDs but also exert the far transfer effect on such people in terms of aspects such as fluid intelligence and academic (reading and mathematical) performance. In other words, WM updating training would somewhat mitigate the symptoms of LDs. Specifically, we predicted differences in ERP amplitude in relation to LDs between the training group and control group, and that the P300 and N160 amplitudes would increase and the P200 amplitude would decrease substantially in the LDs training group.

## Materials and Methods

### Ethics Statement

The study was reviewed and approved by the Ethics Board of Beijing Normal University and the study was conducted in accordance with ethics guidelines of the American Psychological Association. All study participants provided written informed consent prior to the experiment.

### Participants

A total of 65 seventh grade students (31 boys, aged 10–13 years, mean [M] = 11.47, standard deviation [SD] = 0.568) from Beijing Shijingshan Middle School were selected to participate in this study, consisting of 45 children with LDs and 20 without. The children with LDs were randomly assigned to a training group (TLDs group; 15 boys, aged 10–13 years, *M* = 11.45, SD = 0.51) or control group (CLDs group; 12 boys, aged 10–13 years, *M* = 11.47, SD = 0.50). The children with LDs were those who had difficulties in reading and mathematics. The adopted method for determining which children had LDs combined the ability difference comparison method, exclusive method, and screening method. The procedures are detailed as follows: (1) an academic adaptability test (AAT; Zhou, 1991) was administered to all participants. Children whose test levels were ≤ 2 proceeded to the next screening step; (2) teachers familiar with the children were asked to complete the pupil rating scale (PRS; revised). Children whose total scores were <65 proceeded to the next step; (3) the most recent original final examination scores were converted into *Z* scores and children whose Chinese and mathematics scores were <25th percentile were screened out; and (4) Raven’s Standard Progressive Matrices were employed to test the fluid intelligence of each student. Children whose scores were <50th percentile were excluded. The selection criteria for the normal control (NC) group (10 boys and 10 girls, aged 10–13 years, *M* = 11.39, SD = 0.69) were as follows: (1) AAT scores of >2; and (2) *Z* scores for language and mathematics higher than those of the other 25% of students who participated in the final exam. No children in this study exhibited evident visual or sensory impairment, motor difficulties, emotional disorders, social or cultural adaptation problems, or other physical or mental disorders.

### Training Task

**Figure 1:**
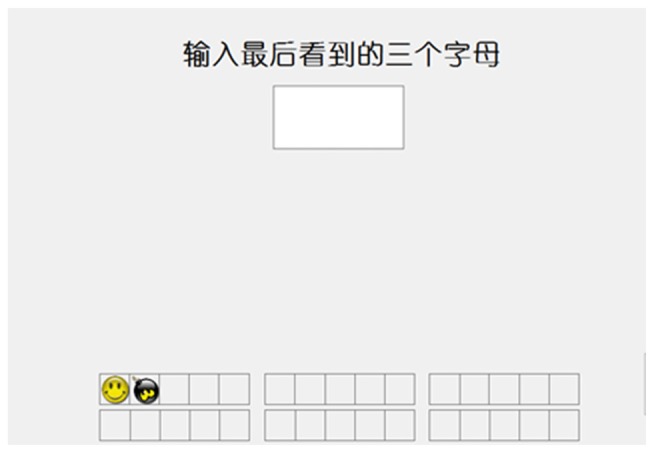
Demonstration of letters running working memory (WM) task.

We used three versions of an adaptive running memory task for training, involving letters, animals and locations (Zhao et al., 2011, 2013). In the letters task (Figure 1), a “+” focus point was displayed in the center of the screen to indicate task commencement. Subsequently, several letters were displayed one by one. The number of letters displayed varied among the different trial types (5, 7, 9, or 11 letters per trial). Each trial type was run an equal number of times in a random order. The participants were required to remember the sequence of the preceding three letters. After being presented with a blank bar on the screen, the participants were asked to use the keyboard to enter the sequence of the preceding three letters. Feedback was provided for each trial. Each letter appeared for 1750 ms; however, the difficulty level continually changed according to the participant’s performance. In this task, each participant completed six blocks and each block contained five trials. If three or more trials were completed correctly, the stimulus intervals were reduced by 100 ms in the subsequent block. By contrast, if two or more trials were completed incorrectly, the stimulus intervals were lengthened by 100 ms in the subsequent block. Each day’s training was based on the preceding day’s performance record for each participant.

**Figure 2:**
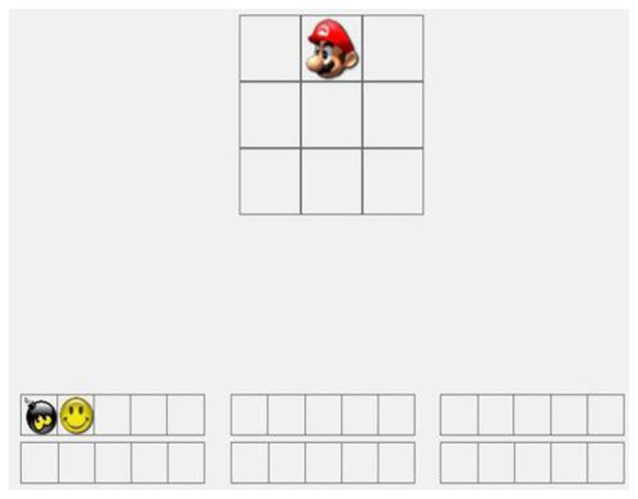
Demonstration of animals running WM task.

**Figure 3:**
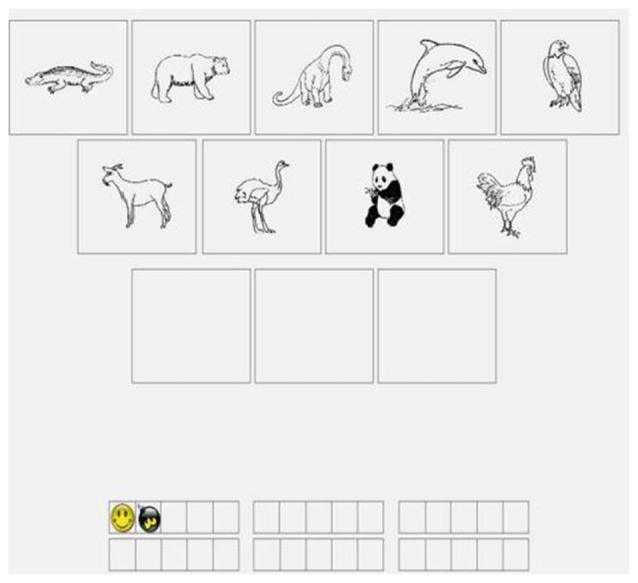
Demonstration of locations running WM task.

The animals task (Figure 2) was identical to the letters task except animal images were used instead of letters. The locations task (Figure 3) was identical to the letters task except visuospatial stimuli were used instead of letters. In each trial, a nine-square grid containing an image of a cartoon face was displayed in the center of the screen. The face could appear in any one of the nine squares. The participants were required to remember the sequence of the preceding three locations. The number of cartoon faces displayed varied among the different trial types (5, 7, 9, or 11 faces per trial). Every trial type was run in a random order. At the end of each sequence, the participants were requested to indicate the sequence of the preceding three locations by clicking in blank squares in a nine-square grid.

### Transfer Tasks

#### 2-Back Task

The 2-back task was employed to measure each participant’s WM updating ability. The task involves a series of random Arabic numerals being displayed one by one. In this study, the participants were required to judge whether each number was the same as the two preceding numbers and respond by pressing a computer key (pressing “1” indicated that the numbers were the same and pressing “3” indicated that they were not). The intervals between stimuli were 1000 ms with a 500 ms delay, during which the participants were required to respond. The entire test consisted of two blocks each with 84 trials. The first two trials in each block required no responses. The ratio of consistent to inconsistent stimuli was 1:1. The reliability coefficient of the 2-back task based on our sample was 0.80.

#### Raven’s Advanced Progressive Matrices Test

Raven’s Advanced Progressive Matrices (APM) test was employed for nonverbal intelligence testing (Jaeggi et al., 2011; Zhao et al., 2011). The test comprised 60 items split into two equal portions based on their serial numbers. The participants were requested to answer the odd-numbered items in the pretest and the even-numbered items in the posttest. Based on our sample, the reliability coefficient of Raven’s APM test was 0.68.

#### Academic Tests

China has no standard academic achievement tests. Midterm and final examinations are generally taken seriously by Chinese students. Test items are designed by teachers who teach the corresponding curricula, and the examinations test students’ mastery of knowledge acquired over a single semester. We applied the children’s language and mathematics scores from the most recent final examination to reflect their pretest academic performance. Their scores from the subsequent midterm examination were applied to reflect their posttest academic performance. Language exams mainly tested reading comprehension (80%) and writing skills (20%), whereas mathematics exams mainly tested calculation (60%) and problem-solving skills (40%). The reliability coefficients of language and mathematics exams were 0.87 and 0.88, respectively.

### Procedure

We employed a double-blind controlled design. All participants were required to complete the 2-back task before and after the training task to enable the near transfer effects to be analyzed, as well as Raven’s APM test and academic tests (language and mathematics) to enable the far transfer effects to be analyzed. The TLDs group was required to complete all three computerized training programs (letters, animals and locations) once per day for 28 days. The CLDs and NC groups were regarded as active control groups; each child in these groups completed unadaptive versions of the same training task. The difference between the two training tasks was that the difficulty of adaptive tasks changed automatically based on a trainee’s performance, meaning these tasks had the potential to improve the trainees’ task performance to the greatest possible extent, whereas the difficulty of unadaptive tasks always remained at the initial level. In addition, compared with the TLDs group, the CLDs and NC groups were required to complete only one-third volume of the unadaptive training tasks everyday.

### ERP Data Collection

Electroencephalograms (EEGs) were recorded using an EEG amplifier (Neuroscan NuAmps40). In the study, participants’ EEG data was recorded during the 2-back task. The sample rate was set to 1000 Hz and a bandpass filter (0.05–100 Hz) was employed during EEG recording. Vertical eye movements were recorded by electrodes positioned above and below the left eye, whereas horizontal eye movements were recorded by electrodes positioned at the outer canthus of each eye. Throughout the recording, the impedance of the electrodes was maintained at <5 kΩ. Remaining artifacts containing a change outside the range of ±100 μV within a period of 50 ms were rejected. Artifact-free EEGs were subsequently segmented into epochs ranging from 200 ms before stimulus onset to 1000 ms after stimulus onset and averaged for each individual participant and condition. Only correct responses were included in the averages, all of which considered at least 50 trials. To attenuate high-frequency noise, the averaged waveforms were filtered using a 30-Hz low-pass filter with 48 dB/octave roll-off.

## Results

### Behavioral Results

SPSS version 19.0 was employed for data aggregation and statistical analysis. Table 1 presents the descriptive statistics of the 2-back task for each group in each session.

**Table 1:** The ACC (%) and reaction time (RT) (ms) of 2-back task of each group in pre- and post-test training (M ± SD).

Group	Index	Pre-test	Post-test
NC (*n* = 20)	ACC	68.96 ± 0.11	74.09 ± 0.12
	RT	777.10 ± 263.88	801.54 ± 251.40
CLD (*n* = 22)	ACC	56.40 ± 0.15	59.77 ± 0.19
	RT	930.84 ± 253.94	937.25 ± 280.92
TLD (*n* = 23)	ACC	57.10 ± 0.14	73.02 ± 0.13
	RT	820.29 ± 286.08	792.57 ± 252.64

A 2 (sessions: pretest and posttest) × 3 (groups: NC, CLDs, and TLDs) repeated measures analysis of variance (ANOVA) was conducted to investigate each participant’s accuracy (ACC) during the 2-back task. The results indicated that the main session effect (*F*_(1,62)_ = 20.005, *p* < 0.001, *η*^2^ = 0.247, *d* = 0.993) was significant and ACC was significantly higher during the posttest than during the pretest. The main group effect (*F*_(2,62)_ = 6.677, *p* = 0.002, *η*^2^ = 0.180, *d* = 0.901) was also significant. *Post hoc* pairwise comparisons results indicated that the ACC in the CLDs group was significantly lower than that in the NC group (*p* = 0.002). No significant differences were observed between the TLDs and CLDs groups (*p* = 0.163) or between the TLDs and NC groups (*p* = 0.233; Bonferroni adjustment for multiple comparisons^1^). Session and group interaction was significant (*F*_(2,62)_ = 4.842, *p* = 0.011, *η*^2^ = 0.137, *d* = 0.781). Although simple effect analysis found no significant differences between the CLDs and TLDs groups in terms of pretest scores (*p* = 0.997), both had significantly lower pretest scores than did the NC group (*p* = 0.009 for the CLDs group; *p* = 0.012 for the TLDs group). Although no significant differences were observed between TLDs and NC groups in terms of posttest scores (*p* = 0.993), both had significantly higher posttest scores than did the CLDs group (*p* = 0.012 for the TLDs group; *p* = 0.008 for the NC group). Although training significantly improved the performance of the TLDs group (*p* < 0.001), comparable results were not observed in the CLDs or NC groups.

A 2 (sessions: pretest and posttest) × 3 (groups: NC, CLDs and TLDs) repeated measures ANOVA was conducted to investigate the participants’ reaction times (RTs) during the 2-back task. The results indicated that the main session effect (*F*_(1,62)_ = 0.001, *p* = 0.973, *η*^2^ = 0.000, *d* = 0.053) and group effect (*F*_(2,62)_ = 2.025, *p* = 0.142, *η*^2^ = 0.004, *d* = 0.063) were nonsignificant. Session and group interaction was also nonsignificant (*F*_(2,62)_ = 0.239, *p* = 0.788, *η*^2^ = 0.004, *d* = 0.065).

A 2 (sessions: pretest and posttest) × 3 (groups: NC, CLDs and TLDs) repeated measures ANOVA was conducted to investigate the participants’ performance in the Raven’s APM test. The results indicated that the main session effect was significant, with posttest performance higher than pretest performance (*F*_(1,62)_ = 22.372, *p* < 0.001, *η*^2^ = 0.285, *d* = 0.996). The main group effect (*F*_(2,62)_ = 0.466, *p* = 0.630, *η*^2^ = 0.016, *d* = 0.122) was nonsignificant. Session and group interaction was nonsignificant (*F*_(2,62)_ = 1.139, *p* = 0.328, *η*^2^ = 0.039, *d* = 0.241). Table 2 lists the Raven’s APM test scores for each group in each session.

**Table 2:** Scores of Raven’s advanced progressive matrices (APM) test of each group in pre- and post-test training.

Group	Pre-test	Post-test
NC (*n* = 20)	101.94 ± 18.14	113.86 ± 14.20
CLD (*n* = 22)	101.16 ± 19.43	106.60 ± 13.84
TLD (*n* = 23)	98.44 ± 15.07	110.38 ± 14.36

To explore the transfer effects of the training, a 2 (sessions: pretest and posttest) × 3 (groups: NC, CLDs and TLDs) repeated measures ANOVA was conducted to analyze the participants’ language and mathematics performance. The results indicated that the main session effect on language performance was nonsignificant (*F*_(2,62)_ = 0.038, *p* = 0.846, *η*^2^ = 0.001, *d* = 0.054), whereas the main group effect was significant (*F*_(2,62)_ = 8.698, *p* < 0.001, *η*^2^ = 0.219, *d* = 0.963). *Post hoc* pairwise comparisons results indicated that language performance in the NC group was significantly higher than that in the TLDs group (*p* = 0.088) and CLDs group (*p* < 0.001); however, no significant differences between the TLDs and CLDs groups were observed (*p* = 0.145). Session and group interaction was nonsignificant (*F*_(2,62)_ = 1.185, *p* = 0.313, *η*^2^ = 0.040, *d* = 0.250). Table 3 lists the pretest and posttest academic performance of each group.

**Table 3:** The academic performance (Z) of each group in pre- and post-test training.

Group	Academic performance	Pre-test	Post-test
NC (*n* = 20)	Language	0.63 ± 0.46	0.58 ± 0.76
	Math	0.79 ± 0.34	0.97 ± 0.28
CLD (*n* = 22)	Language	−0.43 ± 0.72	−0.39 ± 0.83
	Math	−0.55 ± 1.06	−0.36 ± 0.94
TLD (*n* = 23)	Language	−0.12 ± 1.31	−0.22 ± 1.16
	Math	−0.50 ± 0.86	−0.15 ± 0.90

**Figure 4:**
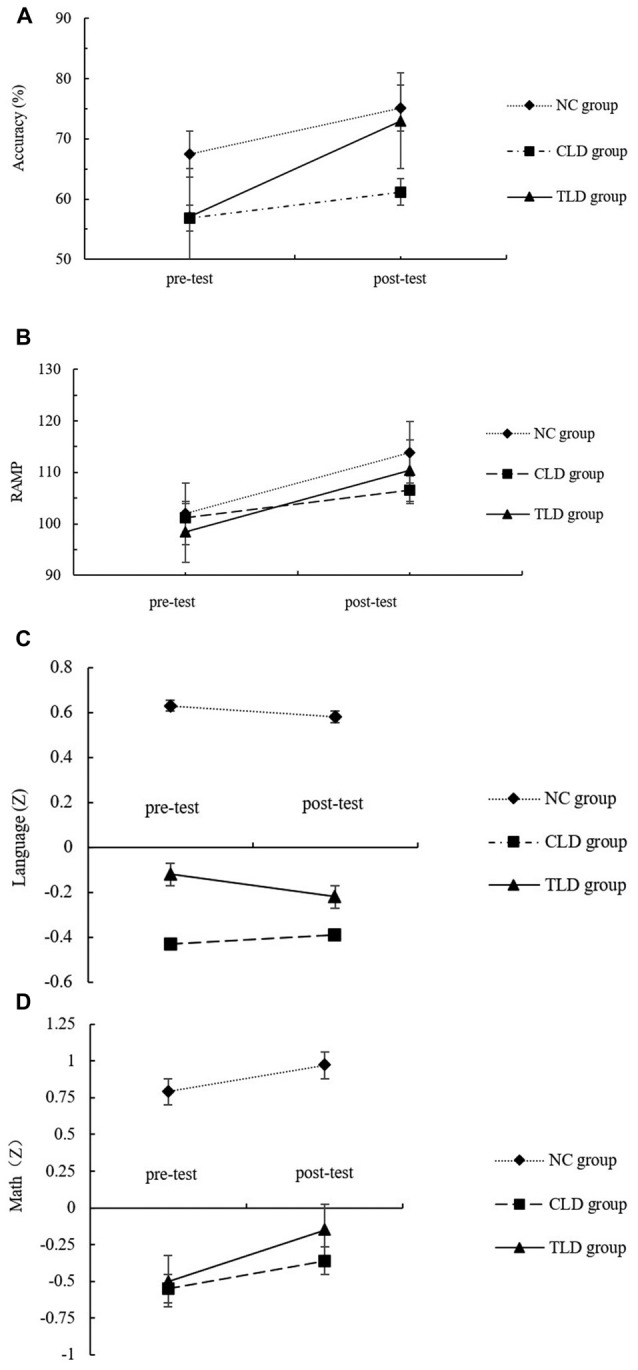
The performance of 2-back task **(A)**, Raven’s advanced progressive matrices (APM; **B**), language grade **(C)** and math grade **(D)** at pretest and posttest for each group.

The main session effect on mathematics performance was significant (*F*_(1,62)_ = 27.346, *p* < 0.001, *η*^2^ = 0.335, *d* = 0.999), indicating higher performance in the posttest than in the pretest. The main group effect was also significant (*F*_(2,62)_ = 14.364, *p* < 0.001, *η*^2^ = 0.305, *d* = 0.998). *Post hoc* pairwise comparisons results indicated that mathematics performance in the NC group was significantly higher than that in the TLDs and CLDs groups (*p* < 0.001 for both) but no significant differences between the TLDs and CLDs groups were observed (*p* = 0.554). Session and group interaction was significant (*F*_(2,62)_ = 6.743, *p* = 0.002, *η*^2^ =0.205, *d* = 0.904). Further analysis indicated that in the pretest, mathematics performance in the NC group was significantly higher than that in the CLDs and TLDs groups (*p* < 0.001 for both) but no significant differences between the TLDs and CLDs groups were observed (*p* = 0.898). In the posttest, mathematics performance in the TLDs group was significantly higher than that in the CLDs group (*p* = 0.004) but significantly lower than that in the NC group (*p* = 0.003). Over the course of the training period, mathematics performance improved significantly in the TLDs group but not in the other groups (Figure 4).

### ERP Results

Following previous research (Zhao et al., 2013) and based on our total average results, we mainly measured the N160 component in the parietal area, P200 component in the frontal area and P300 component in the central area. We measured the peak values of the N160 and P200 components because their amplitudes were apparent. The time windows for the N160 and P200 components were 150–230 and 180–300 ms, respectively. The peak value of the P300 component did not exhibit any large individual differences and several participants did not exhibit any apparent peak values. Therefore, following previous studies on addiction, we measured the average amplitude of the P300 component with a time window of 250–500 ms. Table 4 lists the values of N160, P200 and P300 of each group in pre- and post-test training.

**Table 4:** The values of N160, P200 and P300 (μV) of each group in pre- and post-test training.

Group		Pre-test	Post-test
NC (*n* = 19)	N160 (PZ)	−1.55 ± 2.87	−2.23 ± 3.16
	P200 (FZ)	11.43 ± 5.91	9.60 ± 5.93
	P300 (CZ)	15.67 ± 3.74	16.49 ± 4.26
CLD (*n* = 22)	N160 (PZ)	−0.95 ± 3.06	0.92 ± 2.86
	P200 (FZ)	11.28 ± 4.34	10.98 ± 3.78
	P300 (CZ)	10.06 ± 4.20	9.46 ± 5.25
TLD (*n* = 23)	N160 (PZ)	1.20 ± 3.57	−1.81 ± 2.58
	P200 (FZ)	8.73 ± 3.47	9.47 ± 3.34
	P300 (CZ)	9.89 ± 3.25	14.63 ± 4.42

A 2 (sessions: pretest and posttest) × 3 (groups: NC, CLDs and TLDs) repeated measures ANOVA was conducted to analyze the peak values of the N160 and P200 components and the average amplitude of the P300 component.

**Figure 5:**
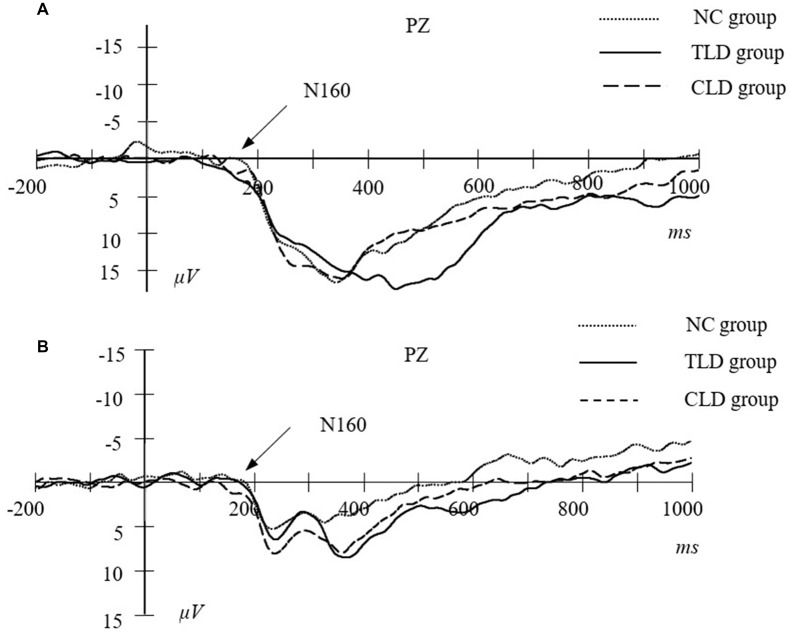
The peak value of N160 in parietal area at pretest **(A)** and posttest **(B)** for each group.

The main session effect at the peak value of the N160 component was significant (*F*_(1,62)_ = 8.886, *p* = 0.004, *η*^2^ = 0.221, *d* = 0.835), indicating that the peak value of the N160 component was significantly higher in the posttest than in the pretest. The main group effect at the peak value of the N160 component was significant (*F*_(2,62)_ = 6.297, *p* = 0.003, *η*^2^ = 0.167, *d* = 0.883). *Post hoc* pairwise comparisons results indicated that the peak value of the N160 component in the NC group was significantly higher than those in the TLDs (*p* = 0.052) and CLDs (*p* = 0.003) groups but no significant differences between the TLDs and CLDs groups were observed (*p* = 0.201). Session and group interaction was significant (*F*_(2,62)_ = 4.626, *p* = 0.013, *η*^2^ = 0.143, *d* = 0.761). Although simple effect analysis revealed no significant differences between the TLDs and CLDs groups in the pretest, both had significantly lower pretest scores than did the NC group (*p* = 0.022 for the TLDs group; *p* = 0.046 for the CLDs group). Although no significant differences were observed between the TLDs and NC groups in the posttest, both had significantly higher posttest scores than did the CLDs group (*p* = 0.006 for the TLDs group; *p* = 0.002 for the NC group). Over the course of the training period, the peak value of the N160 component in the TLDs group improved significantly (*p* < 0.001) but no significant differences were observed between the TLDs group and the CLDs (*p* = 0.975) or NC (*p* = 0.373) groups (Figure 5).

The main session effect at the peak value of the P200 component was nonsignificant (*F*_(1,62)_ = 0.395, *p* = 0.532, *η*^2^ = 0.012, *d* = 0.095), as was the main group effect (*F*_(2,62)_ = 2.028, *p* = 0.140, *η*^2^ = 0.061, *d* = 0.403). Session and group interaction was also nonsignificant (*F*_(2,62)_ = 1.016, *p* = 0.368, *η*^2^ = 0.039, *d* = 0.219).

**Figure 6:**
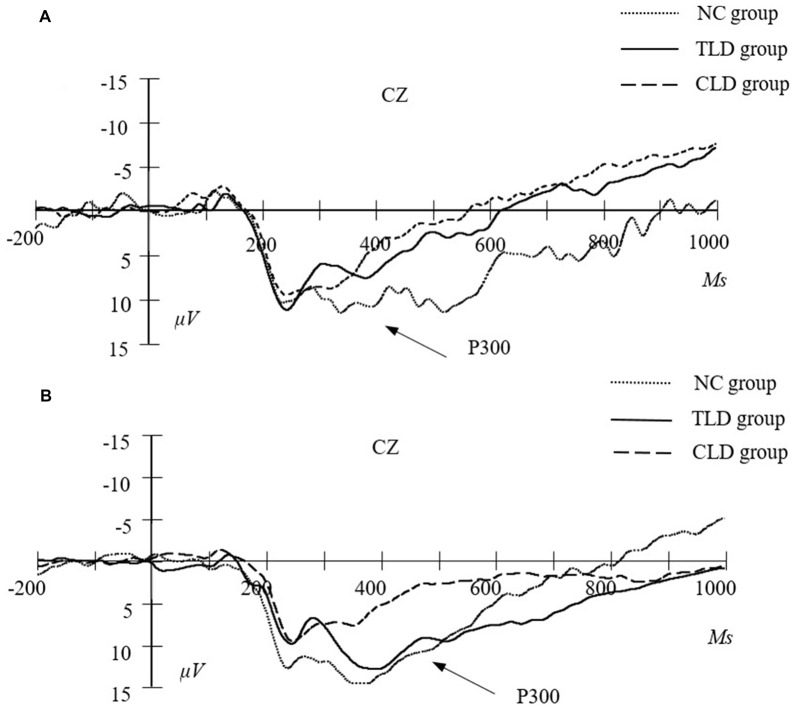
The average amplitude of P300 in central area at pretest **(A)** and posttest **(B)** for each group.

The main session effect at the average amplitude of the P300 component was significant (*F*_(1,62)_ = 5.091, *p* = 0.028, *η*^2^ = 0.079, *d* = 0.602), indicating that the average amplitude of the P300 component in the posttest was significantly higher than that in the pretest. The main group effect at the average amplitude of the P300 component was significant (*F*_(2,62)_ = 22.514, *p* < 0.001, *η*^2^ = 0.433, *d* = 0.99). *Post hoc* pairwise comparisons results indicated that the average amplitude of the P300 component in the CLDs group was significantly lower than those in the TLDs (*p* = 0.039) and NC (*p* = 0.001) groups and the average amplitude of the P300 component in the CLDs group was significantly lower than that in the NC group (*p* < 0.001). Session and group interaction was significant (*F*_(2,62)_ = 4.613, *p* = 0.014, *η*^2^ = 0.135, *d* = 0.759). Although simple effect analysis revealed no significant differences between the TLDs and CLDs groups in the pretest (*p* = 0.998), both had significantly lower pretest scores than did the NC group (*p* < 0.001 for both). No significant differences were observed between the TLDs and NC groups in the posttest (*p* = 0.485) and both had significantly higher posttest scores than did the CLDs group (*p* = 0.003 for the TLDs group; *p* < 0.001 for the NC group). Over the course of the training period, although the average amplitude of the P300 component in the TLDs group improved significantly (*p* = 0.001), it did not differ significantly from those in the CLDs (*p* = 0.643) and NC (*p* = 0.505) groups (Figure 6).

## Discussion

The purpose of the current study was to investigate whether WM updating training could improve WM updating ability in children with LDs. Because impaired learning ability is a core symptom of LDs and studies have demonstrated the benefits of WM training, we investigated whether the effects of WM training could transfer to fluid intelligence and academic performance in children with LDs. ERP results were examined to explore the influence of WM updating training on brain activity in children with LDs.

### Effects of WM Updating Training on WM

Compared with the strategy training, the training we used in this study was belong to “core training” which typically involved repetition of demanding WM tasks that were designed to target domain-general WM mechanisms (Morrison and Chein, 2011). The running memory task we used as the training task better represents the ability to monitor input information and to replace old information, is an index of WM updating ability (Zhao et al., 2011, 2013). In a 2-back task, people were required to judge whether the present Arabic numeral was the same as the two items prior. This means that people have to remember the last three numbers continuously. The 2-back task also used commonly for evaluating WM updating ability (Zhao et al., 2011, 2013; Chen et al., 2017). Because of the similarities in task requirements, the 2-back task and the running memory task that we used as training task may rely on the same cognitive resource so that improvement in the training task contributed to the improvement in the 2-back task (Chen et al., 2017). While no significant improvements in RT were observed in the posttest compared with the pretest, accuracy in the 2-back task increased significantly. Although previous studies have shown that the RT of WM in the 2-back task is an indicator of WM updating ability (Gevins and Smith, 2000; McEvoy et al., 2001; Owen et al., 2005), many other results have suggested that the transfer effect can be observed in terms of task accuracy (Sandberg et al., 2014; Chen et al., 2017). Similar to previous studies (Carretti et al., 2005; Alloway et al., 2006; Wang et al., 2008), our results revealed impaired WM updating ability in children with LDs before WM updating training. Over the course of the 28-day training period in the present study, 2-back task performance in the TLDs group improved significantly compared with that in the CLDs and NC groups, suggesting the occurrence of WM lag in children with LDs. However, in recent years, the results of training completed by children (Thorell et al., 2009; Zhao et al., 2011) and adults (Westerberg and Klingberg, 2007; Jaeggi et al., 2008; Zhao et al., 2013) and people with ADHD (Klingberg et al., 2002), alcohol spectrum disorders (Loomes et al., 2008), and stroke (Westerberg and Klingberg, 2007) have demonstrated that an individual’s WM capability is plastic. According to Gray et al. (2012), WM capability is considerably impaired in children with LDs or ADHD compared with children without these conditions. Their study was the first to provide individuals with LDs or ADHD with Cogmed WM training and mathematics training. The researchers observed that the WM training program yielded a considerably larger improvement in verbal WM capability than did the mathematics training program and that this effect could transfer to visuospatial WM capability. Another study observed that college students with LDs or ADHD exhibited considerable improvement in attention deficit and cognitive difficulties after 5 weeks of Cogmed WM training (Gropper et al., 2014). The Cogmed training program is aimed at improving general WM capability by influencing each WM component (Klingberg et al., 2005; Holmes et al., 2009). The current study provided evidence that the effects of “pure” training on WM updating ability are comparable to those of the Cogmed program.

### Effects of WM Updating Training on Academic Performance

Compared with short-term memory, WM plays a more influential role in children’s academic performance (Peng and Fuchs, 2014) because many academic tasks involve multiple processes with intermediate solutions that children need to remember as they proceed through the tasks (McKenzie et al., 2003; Cain et al., 2004). The most prominent symptom of LDs is poor reading and mathematics abilities, both of which directly influence academic performance (Swanson and Beebe-Frankenberger, 2004; Gathercole et al., 2006; Geary et al., 2007). Core training seeks to produce increased WM capability by focusing on the strengthening of domain-general WM processes. If these WM processes are indeed strengthened, this approach would yield improvements not only on tasks similar to those used in training, but also, on more disparate cognitive measures (Morrison and Chein, 2011). Following previous related studies, our study investigated the transfer effect of WM updating training on academic performance in children with LDs. Similar to the results of previous studies (Kroesbergen et al., 2007; Passolunghi et al., 2007; Swanson and Kim, 2007; Witt, 2011; Alloway et al., 2013; Dahlin, 2013), the children with LDs in the present study exhibited lower language and mathematics performance than their counterparts without LDs. Over the course of the 28-day training period, mathematics performance in the TLDs group improved significantly compared with that in the CLDs and NC groups, suggesting that this outcome was the result of training rather than self-development. Other studies that have employed the Cogmed program to train WM in children and college students with LDs have not reported any such improvements in mathematics performance (Gray et al., 2012; Gropper et al., 2014), indicating that compared with the Cogmed program, the WM updating training method employed in the current study might exert a stronger and more direct effect on mathematics performance.

We observed no language improvement in our study, which was inconsistent with the findings of Loosli et al. (2011), who trained children without LDs aged 9–11 years for 10 days through a WM span task and observed a transfer effect on reading ability. Based on the hypotheses that transfer occurs if the criteria and transfer tasks initially engage similar processes and that brain circuits predict overlapping activity before training (Dahlin et al., 2008), a possible explanation for the outcome observed by Loosli et al. (2011) is that updating ability might be more closely related to mathematical ability than reading ability. In mathematical learning, updating operational symbols is more crucial than applying basic operational rules. Therefore, updating training might be a direct and effective method for improving mathematical ability. Another possible explanation is that improving language ability requires more time than does improving mathematical ability. This could serve as a target of investigation for future studies.

### Effects of WM Updating Training on Fluid Intelligence

Fluid intelligence is a complex ability that enables an individual to adapt their thinking to new cognitive problems and situations (Carpenter et al., 1990; Jaeggi et al., 2008). Many studies have suggested that WM capability and fluid intelligence share neural networks in the lateral prefrontal and parietal cortices (Kane and Engle, 2002; Gray et al., 2003; Owen et al., 2005; Jung and Haier, 2007; Jaeggi et al., 2008). Several studies have reported that WM can be used to predict fluid intelligence (Jaeggi et al., 2008; Engle, 2010; Zhao and Zhou, 2010). Based on the results of these studies, the possibility of improving fluid intelligence through WM training has been investigated (Jaeggi et al., 2008; Sternberg, 2008; Zhao and Zhou, 2010; Zhao et al., 2013). Whether WM updating training is able to improve fluid intelligence has been strongly debated in recent years (Harrison et al., 2013; Melby-Lervåg and Hulme, 2013; Redick et al., 2013; Au et al., 2014; Bogg and Lasecki, 2015). Although many studies have revealed positive effects of WM updating training on fluid intelligence (Jaeggi et al., 2008; Zhao et al., 2011; Chen et al., 2017), the Raven’s APM performance in the TLDs group in the present study did not exhibit considerable improvement after training compared with that in the CLDs and NC groups. This outcome indicated that WM updating training did not exert a far transfer effect in children with LDs, supported the viewpoint that WM updating training couldn’t improve fluid intelligence, which was in agreement with the findings of many previous studies, where various training programs have exhibited limited efficacy in terms of improving fluid intelligence (Conway and Getz, 2010; Shipstead et al., 2010, 2012; Morrison and Chein, 2011). We offer three explanations for this result. One is that the training effect is delayed; Holmes et al. (2009) revealed that although children with LDs who received WM training exhibited no immediate improvements in fluid intelligence, a follow-up test conducted 6 months after the training revealed such improvements. The second explanation is that the training effect on fluid intelligence is dependent on the degree of improvement exhibited by participants in the WM task, as shown in previous studies (Jaeggi et al., 2011). Gevins found that subjects with higher IQ exhibited considerably lower RTs in the 2-back task, meaning that RT is a major indicator of IQ (Gevins and Smith, 2000). Based on our results, we found that RT in the 2-back task had not improved after training in the TLDs group compared with the CLDs and NC groups. The last explanation is that, whereas WM and fluid intelligence are highly related and it would be easy to conclude that they reflect the same cognitive mechanism, they are separable constructs (Heitz et al., 2006). The correlation between WM and fluid intelligence is at nearly the same level as weight and height in humans (*r* = 0.47 in the latter case; Freedman et al., 1998); while nobody would assume that making someone taller would also make them heavier. The results suggested that WM and fluid intelligence were different hypothetical constructs and that an intervention that improve WM may have no effect on fluid intelligence (Harrison et al., 2013). Based on these findings, we recommend verification through further research.

There was also an interesting result that, although there was no significant improvement, the fluid intelligence of participants in each group seemed to be improved over time. It indicated that the fluid intelligence between LD and normal children were all plastic and all can be improved by the WM updating training, which is supported by Chen and Li (2007) that WM updating has been found to mediate the association between age and fluid intelligence.

### Effects of WM Updating Training on Brain Activity

ERPs are electrical activities locked to specific task events or responses. They offer high temporal resolutions (within a range of milliseconds) for neural processes underlying behavioral performance (Banaschewski and Brandeis, 2007; Liu et al., 2014). We adopted ERPs to examine the effect of WM updating training on brain activity in children with LDs. The results indicated that the average amplitude of the P300 component in children in the TLDs group substantially increased over the course of the training, eventually approximating that in children without LDs. The P300 component has been regarded as a valid index for WM updating ability (Gevins et al., 1996; Rousselet et al., 2008). In the context-updating model, when information is presented, the brain responds and applies the new information to the current situation, thereby forming new representations to replace old ones. The brain should adjust current information based on the continuously changing environment (Donchin, 1981; Donchin and Coles, 1988). Studies have suggested that individuals with high cognitive abilities exhibit a higher average P300 amplitude. Furthermore, studies on cognitive aging have provided evidence that the average P300 amplitude decreases with age (McEvoy et al., 2001). The results of the current study indicated that training can improve the WM updating capability of children with LDs, which agrees with the findings of Zhao et al. (2011), who observed an increase in the average P300 amplitude in adults after 20 days of WM updating training.

The results of the current study indicated that the peak N160 value in the TLDs group increased significantly throughout the training period. The N160 component was considered a valid index for visual recognition processing and the peak N160 value was proportional to the strength and effectiveness of a target’s visual recognition (McEvoy et al., 2001). Moreover, the component in the central area represents the cognitive control function (Daffner et al., 2000; Suwazono et al., 2000). In our study, the increased peak N160 value after training suggested that WM updating training can improve the strength and validity of an individual’s stimulus recognition. This indicates that the effects of training are exerted at the perception stage, thereby facilitating improvement in cognitive control function and updating ability.

No decrease in P200 amplitude in the TLDs group was observed after the WM updating training. The P200 amplitude evoked from the frontal area reflects the inhibition of irrelevant information and the ability to attend to the target stimulus (McEvoy et al., 2001; Owen et al., 2005; Zhao et al., 2013). This suggested that training could not improve aspects of the inhibitory capacity such as updating in children with LDs. The results of this study differed from those of Zhao et al. (2013), who conducted 20 days of WM updating training on healthy adults and observed a considerable decrease in P200 amplitude in the frontal area. In addition, studies have shown that LDs can cause inhibition deficits (Wang et al., 2012; De Weerdt et al., 2013; Liu et al., 2014). Special training for the inhibitory capacity of WM in children with LDs could be useful in influencing the P200 component.

## Conclusion

WM capability is plastic in children with LDs. These children exhibit WM deficiencies that can be mitigated through WM updating training. The effects of WM updating training can transfer to cognitive functions such as mathematical ability. Changes in brain activity are associated with improved cognitive control and WM updating ability, both of which are associated with WM updating training.

## Author Contributions

HZ and RZ were in charge of framing the questions, conducting the study and writing up the results. XC and LM helped with data collection. LC was in charge of final revision and modification.

## Conflict of Interest Statement

The authors declare that the research was conducted in the absence of any commercial or financial relationships that could be construed as a potential conflict of interest.

## Footnotes

^1^The Bonferroni correction was used for the corrections of the multiple comparisons in this study.
